# Investigation of an Intelligent System for Fiber Optic-Based Epidural Anesthesia

**DOI:** 10.1155/2014/437679

**Published:** 2014-03-20

**Authors:** Cihun-Siyong Alex Gong, Chien-Kun Ting

**Affiliations:** ^1^Department of Electrical Engineering, School of Electrical and Computer Engineering, College of Engineering and Portable Energy System Group of Green Technology Research Center, Chang Gung University, Taoyuan 333, Taiwan; ^2^Department of Anesthesiology, Taipei Veterans General Hospital and National Yang-Ming University, Taipei 112, Taiwan

## Abstract

Even though there have been many approaches to assist the anesthesiologists in performing regional anesthesia, none of the prior arts may be said as an unrestricted technique. The lack of a design that is with sufficient sensitivity to the targets of interest and automatic indication of needle placement makes it difficult to all-round implementation of field usage of objectiveness. In addition, light-weight easy-to-use realization is the key point of portability. This paper reports on an intelligent system of epidural space identification using optical technique, with particular emphasis on efficiency-enhanced aspects. Statistical algorithms, implemented in a dedicated field-programmable hardware platform along with an on-platform application-specific integrated chip, used to advance real-time self decision making in needle advancement are discussed together with the feedback results. Clinicians' viewpoint of improving the correct rate of our technique is explained in detail. Our study demonstrates not only that the improved system is able to behave as if it is a skillful anesthesiologist but also it has potential to bring promising assist into clinical use under varied conditions and small amount of sample, provided that several concerns are addressed.

## 1. Introduction

Ever-increasing demand for advance on clinical technologies is driving a revolution in information and communications engineering to enable effective and sustainable solutions to pressing problems in medicine. For epidural anesthesia, it is believed that an experienced physician will still cause serious complications due to the failure of epidural needle placement [1–3]. There is about 10% of epidurals fail rate for a skillful anesthesiologist [4, 5]. As a result, substantial improvement in methodology would require concerted, long-term actions with interdisciplinary collaborations among scientists, engineers, medical researchers, and practitioners.

The lack of a truly efficient object-based approach for quantification will result in unpredicted quality and turns out to be fear of failure for anesthesiologists who understand the resulting medical complications. Several techniques have been demonstrated so far in the literature to improve the clinical practice since the possibly reported invention of the first lumbar puncture in 1885 [6]. However, the intrinsic physical properties of them limit their practicalities. The techniques of today reflect a technological revolution for which the ongoing progress is accepted and adopted by most of the anesthesiologists from time to time. We briefly introduce the techniques as follows: Young et al. [7] proposed using electrical method to measure the impedances stemming from the degree of close texture in tissues. The significantly high impedance of the epidural space (ES) can be considered as an index to differentiate it from others. The drawback of this method might be its electricity which is the physiological nature of the human being for neural transmission (disturbance!). The inherent “negative pressure” characteristics of the epidural space can also be used to identify it from the dura mater and ligamentum flavum (LF).

The commonly seen “loss-of-resistance” technique is difficult to learn for anesthesia residents [8]. Moreover, several postoperative ill-effects such as the pneumocephalus [9, 10], for instance, made it not preferred, mainly due to the over-limitation medium. The use of fluid as a medium may dilute the concentration of anesthetics and potential obscureness for differentiation in the cerebrospinal fluid leaked [11, 12]. Ultrasound has been used to characterize properties of the tissue-tissue interface with respect to accuracy and safety of the operation [13]. Despite the advantage that it is a relatively new area of technology, its bulky size makes it impractical, turning out to be of academic interest only.

Our team has demonstrated a novel optically guided method [14, 15] to achieve a quality performance as an experienced physician did when used in epidural blockade. An prototypical recognition system used only single discriminant analysis method to guide epidural placement and reduce physician error was previously developed [16]. There is, however, still room to be improved. In current study, we present an improved intelligent recognition system (i-RS) with hardware description language to build a real-time decision making which helps the anesthesiologist in epidural space identification. We also performed animal study to test and validate the performance of performance.

## 2. Methods

### 2.1. Novel Optical Guided Needle Design

**Figure 1:**
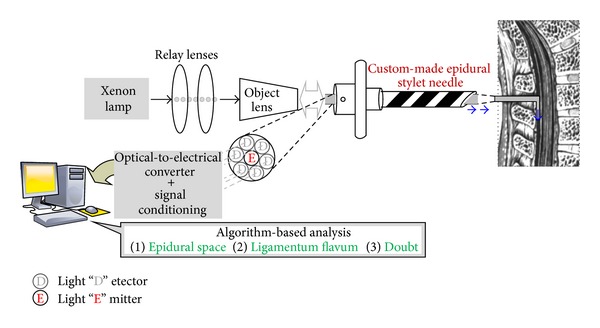
Illustration of fibre-optical regional anesthesia technology.

In our previous study, our team has investigated, for the first time, a breakthrough in assisting epidural space identification technology by using a special designed stylet containing optic fibers. The custom-made stylet contained one center fiber which was used to deliver light to the tissues and six surrounding fibers which were used to collect the reflective signals from the tissues. With Xenon lamp and a monochromator, tissue properties associated with the epidural space of porcine were found* in vitro* (Figure 1). Two wavelengths 532 nm and 650 nm were selected from the* in vitro* study and used in the study of animal models. Only the latter was demonstrated to be useful in the* in vivo* study. The area under curve of receiver operating characteristic for the red light was up to 89%, implying its success in the living porcine model as compared with that of its counterpart. It provided most information in our later discrimination analysis [14, 15].

### 2.2. Portable Intelligent System for Optical-Technology-Based Epidural Space Detection

After proper optoelectronic transformation and signal conditioning through the front end, the data representing the reflected portion of the light emitted were postprocessed and analyzed by means of our self-designed software at personal computer. The software-based analysis provides a decent ideal of discrimination and application. It is, however, not good for practice, due to its cumbersome movement in size.

**Figure 2:**
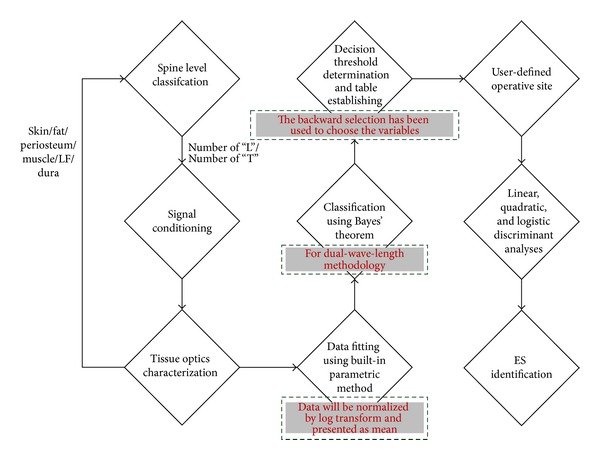
Flowchart of stand-alone hardware-accelerated implementation using ICs.

Modern operating theaters are full of electrical wires and therefore could not accommodate themselves to having an unrealistic system. In addition, it is not good for real-time operation by the nature of the software. Moreover, the intelligent system could correctly detect the epidural space only when the two categories of tissue layers (EF and LF) could be distinguished from each other by the built-in algorithm. This means that the most important thing toward acceptable performance is to find “decision” thresholds in a way that is real time as there are slight variations at different spine levels. Apparently, there is still room for improvement on the operating procedures of such a new technology. An improved methodology is proposed in Figure 2. To realize a design towards portable solution for the sake of reduction of the space occupied by the electrical wires and instruments in the operating theaters, our new-generation system was built using Stratix III 3S150 device (Altera) with a full-custom application-specific integrated circuit (ASIC) which was implemented using the mixed-signal IC design flow with a 0.35 *μ*m CMOS process and fabricated by semiconductor foundry except the first three blocks in the flowchart.

Hardware-efficient methodology is a key to real-time processing. As a result, the backward selection has been adopted since the elimination procedure is, to the best of our knowledge, the simplest of all the counterparts of selecting the best subset of predictors. Its feature of realization without special software is attractive in terms of cost-effective miniaturization and low-power viewpoints. To compute the adopted procedure, the Bayes formula was employed and implemented in the hardware. The backward selection ends up finding proper decision thresholds for postprocessing. The resulting thresholds from the chosen variables, followed by the “user-defined operative site” dialog box, were used for the classifiers.

### 2.3. Animal Study

**Figure 3:**
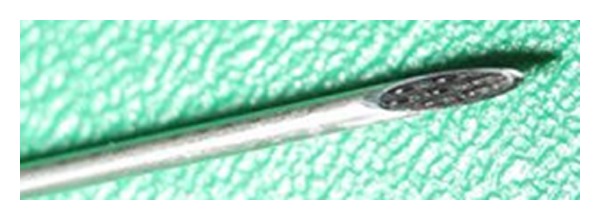
Fibre-optical needle tip showing the fibers glued together.

After approved by Institutional Animal Care and Use Committee of Taipei Veterans General Hospital, ten Duroc and Landrance, Chinese native pigs were enrolled into this study. All pigs in the study were anaesthetized with tiletamine-zolazepam at a dosage of 5 mg/kg and intubated. The anesthesia was maintained with isoflurane (1.5–2%) during the whole operation. The pigs used in this study were weighted around 25 kg. The pigs were placed in the left lateral position with standard monitors. The puncture was carried out with a 17-gauge Tuohy needle (Arrow, USA) which contained a special designed optical stylet. It was inserted into the back of pig, from the low lumbar to middle-thoracic region to collect the optical reflection data. Each pig received 10–12 punctures from its thoracic to the lumbar region bilaterally. While puncturing, the needle advanced in a slow and constant speed to avoid accident dural puncture. Aiming at the porcine spinal cords, a hollow epidural needle that contains a custom-made stylet with seven fibers inside performs catheterization, which can be observed from Figure 3. The six peripheral fibers of the central counterpart receive reflected portion of a 650 nm emitted light from the central fiber to the encountering tissues under needle advancement, while at the same time transferring it to the core of our discriminant system.

### 2.4. Statistics

Despite the results shown in our early studies [16] indicating that “LDA was the best fit to discriminate between the epidural space and ligamentum flavum in our model,” the logistic discrimination was also implemented in our portable hardware platform to pursue higher accuracy of the optical needle placement system. Linear discriminant analysis (LDA) deals with the data by assuming that the conditional probability density functions are normally distributed while logistic discrimination is without this assumption. Bayes' theorem (Formula 1) was applied as a classification procedure for both discriminant methods. Data derived from the reflective signals were normalized by logarithmic transformation. A timely decision on indication of the arrival of the needle tip would be made as long as a conclusion was given by mixed analyses among the three types of discriminant methods with cross-validation:

(1) $$\begin{array}{c} p\left(k\;|\;x\right)=\text{Pr}\left\{C=k\;|\;X=x\right\}=\frac{\pi_{k}p_{k}\left(x\right)}{\sum_{l=1}^{K}\pi_{l}p_{l}\left(x\right)}. \end{array}$$

Formula 1: when *X* = *x*, we can have the* k*th group's posterior probability *p*.

In addition, a category of uncertainty associated with the indeterminate observations has been added to the random access memory of our system as a hardware look-up table (LUT) by setting a minimum acceptable posterior probability after several rounds of clinical trials. It turns out to be a safe decision threshold, automatically adjusted by the on-chip finite state machine, to maximally alleviate the medical complications stemming from the advancement of epidural needle. The new system using the proposed methodology features light weight, enhanced operation speed, and qualified intelligence.

**Figure 4:**
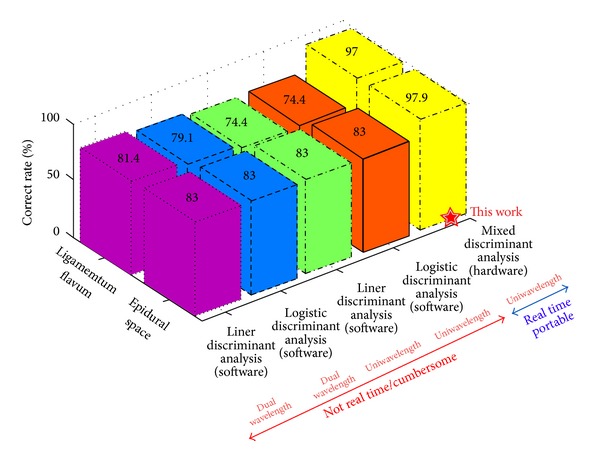
Performance comparison.

The reflective data collected from the epidural space and ligamentum flavum were fitted using the parametric methods to fulfill analysis from two classes of the optical signals from the two tissues of interest. Those were derived from four kinds of combinations, “dual (532 nm and 650 nm)-wavelength linear discriminant analysis,” “dual (532 nm and 650 nm)-wavelength logistic discriminant analysis,” “uni (650 nm)-wavelength linear discriminant analysis,” and “uni (650 nm)-wavelength logistic discriminant analysis,” each of pairs of reflective signals from the ligamentum flavum and epidural space. The comparison method is shown in Figure 4. A linear mixed model was also used to compare the signals of ligamentum flavum and epidural space at different vertebral levels, with random effects to account for the correlation in each measurement from the same pig. All statistical analyses were performed with SAS software (V9.2; SAS Institute Inc., Cary, NC, USA).

## 3. Results and Discussion

**Figure 5:**
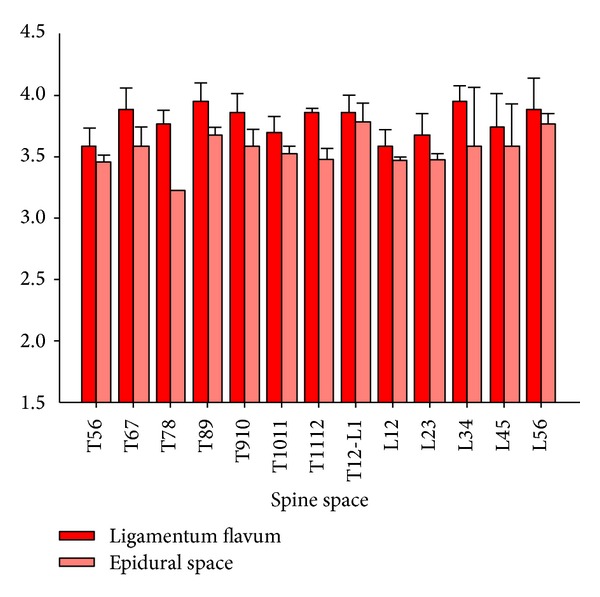
Reflected strength in 650 nm emitted light for different spine levels.

There were totally 216 records of reflective data collected, where 112 (52%) came from the epidural space and the rest of the data were from the ligamentum flavum.  Figure 5 shows the data after careful spine level classification followed by the signal conditioning and tissue optics characterization. The unit of the vertical axis in Figure 5 is none as it represents the “relative” readings obtained by dividing the emitted power by the reflected counterpart. Statistically, we observed that there were no significant differences for different puncture sites (supported by a linear mixed model analysis of a total of dozens of needle insertions) so the low variability of our data supports the feasibility to use tissue optics as a discrimination tool for epidural needle placement.

**Figure 6:**
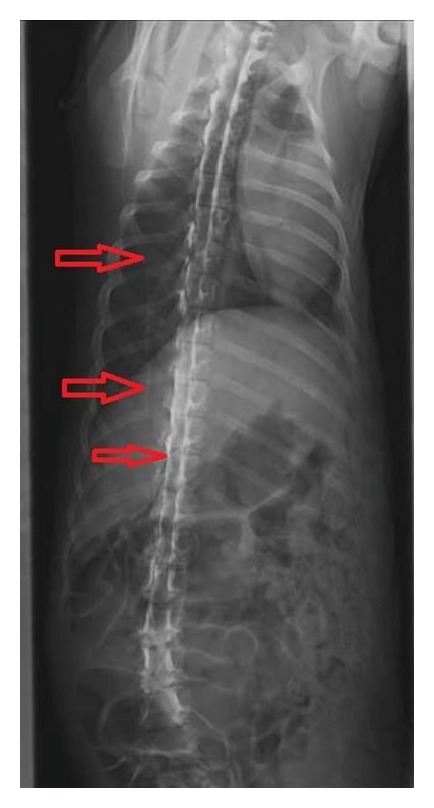
Proof of concept in a successful epidural needle placement using the proposed real-time hardware system.

The resulting “mean” calculated from the data from each of 216 pairs of reflective signals from the ligamentum flavum and epidural space can be used to describe “population.” The leave-one-out cross-validation method has been used to minimize the ill-effect caused by classification error and the radiography with contrast medium was used to confirm proper epidural needle placement (Figure 6).

Our comparison demonstrates that this newly developed system was not only best in performance, but real time in epidural space identification. The results also suggest that the uniwavelength design is not a problem as long as the sample numbers are sufficiently large and the mixed analyses methodology is adopted. Note that the correct rate was deduced from corresponding “sensitivity” of target. The measured average power consumption of the system was about 400 mW for different operations, providing supporting evidence for its main objective portability.

Acknowledge of epidural blockade is limited by the slow onset, high failure rate, and neurological complications. The success lies on how to identify the epidural space exactly. Unlike the traditional LOR technique that depends on subjective and unreliable detection of the mechanical resistance to injection of air or saline, this intelligent system using optical detection can help the anesthesiologist to make decision whether epidural space is reached or not with reproducible and quantitative parameters. In this study, we successfully demonstrate the portable intelligent system with an FPGA platform implementing our i-RS algorithm and a CMOS chip in charge of the signal acquisition and conditioning at its front end. It was built for decision making during epidural needle insertion and provided real-time information for locating the position of the tip of epidural needle. Anatomically, the ligamentum flavum lies directly over the epidural space, so that accurate epidural placement was possible if either the ligamentum flavum or epidural space could be identified [15, 16]. As was described in our previous study [16], the i-RS algorithm could do only two possible decisions (binary), each based upon the certainty of class identification. If the ligamentum flavum is identified, the needle is inserted deeper and if the epidural space is identified, the optical stylet is removed and the catheter is inserted.

The epidural space contained fat, blood vessels, and lymphatic sand nerve roots. During the needle insertion, a small volume of blood, tissue, or both could have remained on the bevel surface and altered the reflective optical signals. These conditions, especially the blood, can have a big influence on the results and may account for the notable differences seen in the* ex vivo *and* in vivo *studies. We found the wavelength that seems most optimal in the* ex vivo *study and failed to satisfy the receiver operating characteristic curve requirements* in vivo *in both the current and previous studies [15]. Future i-RS algorithms should try minimizing the inference of blood by changing to a new wave length such that it could potentially provide a more accurate estimation of the reflective signals and therefore differentiate ES from LF precisely.

Optical methods show great promise for needle localization because of their compact nature [17]. Tiny optical instrument could allow micrometer precision in locating the epidural space. Our system demonstrates a brand new proof of concept combining miniaturized hardware integration with intelligent hardware-accelerated algorithms to achieve operation aid for epidural anesthesia. Modern optical design with directly interfaced integrated circuits and systems creates minimally invasive possibility at extremely restricted places. The FPGA-implemented prototype is just first trial. Despite its flexibility in rapid prototyping, the routing caused by the gates of FPGA array turns out long critical paths and high fan outs, resulting in power wasted. Moreover, the demand on light-weight portability is compromised as a result of its volume, even if we have made great improvement on overall design as compared with the state of the arts [18, 19].

The limit can be resolved by means of the full-custom design using the system-on-a-chip (SoC) methodology [20]. The SoC methodology has been in part adopted in the current design. It will be one of our goals in the near future to realize the entire system on a single IC. Another limitation concerns the increase in the number of samples. In addition, aside from the continuous enhancement in the efficiency of successful needle placement rate for the algorithms, the degree of maturity of the novel technique could not be judged without consideration for the appearance of the samples. In addition, the gender identity of tested sample might affect the results significantly. All the factors should be studied in depth.

## 4. Conclusion

In line with the trend of concurrent demand for operation time reduction and failure risk reduction in the regional anesthesia, our goal is to provide both scientific and objective tool that is efficient to the physicians. For the medical community, a wide range of experience along with many clinical influences, such as the type of build, age, and sex distinction, relegates the physician-guided method to a blind technique. Despite the fact that there have been many techniques to assist the anesthesiologists in correctly performing the regional anesthesia, none of them suffers any limits. They are yet to report a sufficient success rate. The real-time optically reflective signals obtained in living porcine models by using the fibers inserted in an epidural needle have been demonstrated to possess capability to achieve higher rates of successfully guiding epidural needle placement, provided that the sample numbers are sufficiently large and our mixed analyses methodology is adopted. The results allow the proposed system to be improved with the sample numbers associated with the improvement on the overall sensitivity and specificity of the discriminant methods. To avoid puncturing (inserting the catheter) causing serious medical complications, a category of uncertainty has been added into the real-time system to be functional for safe threshold under Bayes' theorem. Our next step is to continuously give consideration to increasing the training set, decreasing the power dissipation, and shrinking the current design.

Unfortunately, even though the efforts we have made to improve the performance of our technique, we can only have limiting sample size to train our mixed analyses with LDA and logistic discrimination as a result of the difficulty in the time-consuming experiment. The proposed technique would not be matured if the developed algorithm is still rudimentary. To deal with this problem, we have considered another approach to improve performance of our intelligent system in the oncoming study. A semisupervised classifier design that simultaneously learns from large amount of unlabeled data, together with labeled data, is another approach to build better classifier [21].

For example, a hybrid generative/discriminative approach is one of the methods to do semisupervised classifier [22]. For this approach, the intelligent recognition system not only learns from known labeled sample in our original study but also learns from new unlabeled samples during clinical practice. Therefore, the performance of the intelligent system will be improved as gathering large amount of data. We hope that, by leveraging the proposed fiberoptic-assisted technique, we can create significant benefit to the physicians. The present system making timely decision behaved like a well-trained anesthesiologist. It is possible to become a promising tool that is more intelligent in the near future.
